# Determinants of pre-adolescent girls’ sport performance in three-year swimming training

**DOI:** 10.3389/fspor.2025.1710646

**Published:** 2025-12-03

**Authors:** Mariusz J. Kuberski, Agnieszka Musial, Piotr Krużołek, Michalina Błażkiewicz, Jacek Wąsik, Jan Konarski

**Affiliations:** 1 Uniwersytet Jana Dlugosza w Czestochowie, Częstochowa, Poland; 2 School of Biological and Behavioural Sciences, Queen Mary University of London, London, United Kingdom; 3 Sports Championship Secondary School Complex, Racibórz, Poland; 4 Akademia Wychowania Fizycznego Jozefa Pilsudskego w Warszawie, Warsaw, Poland; 5 Akademia Wychowania Fizycznego im Eugeniusza Piaseckiego w Poznaniu, Poznań, Poland

**Keywords:** swimming, front crawl, longitudinal study, girls, measurement

## Abstract

**Aims:**

The aim of the study was to identify which anthropometric, physiological, and respiratory parameters are the most important determinants of 400 m front crawl swimming performance among prepubescent, non-elite female swimmers.

**Materials and methods:**

The study group consisted of 14 swimmers (mean biological age at baseline: 10.52 ± 0.37 years; body mass: 34.99 ± 2.77 kg; height: 146.00 ± 3.05 cm). The study was conducted over three years. The swimmers followed endurance training recommended by the British Swimming Federation. Every six months, the following parameters were measured: percentage of body fat, anthropometric variables, aerobic and anaerobic capacity, respiratory parameters [vital capacity [VC], forced expiratory volume in 1 s [FEV_1_], forced vital capacity [FVC]], and a 400 m front crawl swimming test.

**Results:**

After accounting for multicollinearity, the most influential factors determining 400 m front crawl performance were foot dimensions, VC and % body fat. Other somatic and physiological predictors had a less of an impact on swimming performance.

**Conclusions:**

The 400 m front crawl performance among prepubescent girls is not determined by aerobic capacity but rather by foot length and respiratory indicators.

## 1 Introduction

The beneficial effects of swimming on the human body are well established. It has been shown that swimming from an early age helps correct postural defects, has a positive influence on the cardiovascular system, and provides a foundation for the prevention of obesity (1, 2). It should also be emphasised that, in order to achieve success in international competition, athletic training needs to begin before the onset of puberty (3). At the same time, swimming training must not interfere with the natural development of the body (4). The steady improvement in swimming performance and the frequent breaking of world records across different distances and styles compel coaches and athletes aiming to compete at the international level to seek increasingly innovative training solutions. This tendency is visible at every stage of training. The pressure to achieve results at international, national, and even local levels is so considerable that, when faced with the choice between carefully designed training methods and simply increasing training mileage, the latter option is often preferred. Excessive biological strain placed on children (in extreme cases) may, however, inhibit their sporting development and prevent them from realising their full potential in senior competition. Exploring new training methods and understanding the contribution of specific factors to swimming performance in particular strokes and distances therefore appear to be more appropriate ways of enhancing performance without disrupting the child's natural biological development (5–7). Many coaches select children who possess specific anthropometric characteristics (e.g., greater body height, long upper and lower limbs, narrow hips). However, such selection does not always translate into efficiency in swimming performance in later years (8). It is also important to note that in late childhood (ages 9–11), training should support the development of motivation and self-awareness. Emphasis should be placed on enjoyment, skill acquisition, and the development of motor abilities, while minimising situations focused solely on competitive success. This developmental stage is characterised by emotional lability, mood fluctuations, and the experience of strong positive and negative emotions. Children at this age also begin to learn how to regulate their emotional states, to take account of peer opinion, and to develop psychological resilience (9). In practical terms, promoting aerobic capacity and muscular strength in young athletes is essential for supporting motor learning, enhancing physical fitness, and consequently improving performance. Such an approach contributes not only to better health and well-being, but also to reducing the risk of injury at an early age and increasing the likelihood of achieving elite results in adulthood (10, 11). Sports performance depends on numerous factors, among which genetic predispositions—including innate morphological characteristics—as well as appropriately adjusted training loads. In swimming training for prepubescent children, it is essential to take account of differences in morpho-physiological traits that change with ontogenetic development. These differences include bone growth, muscle and fat distribution, motor skills, and the functioning of the respiratory and cardiovascular systems. One of the most important recommendations in training young swimmers is therefore to adapt training programmes to the athlete's biological age (12). At present, freestyle has the widest range of competition distances: 50 m, 100 m, 200 m, 400 m, 800 m, and 1,500 m. In such type of competition, virtually all swimmers adopt the fastest technique, the front crawl. It is also the stroke used to cover the greatest proportion of training distance by young swimmers, irrespective of emerging specialisation. Consequently, the front crawl dominates over other techniques and is most frequently the focus of research in swimming. There is, however, no consensus as to which variables most strongly influence long-distance front crawl performance in prepubescent children. Some studies suggest that somatic build is paramount, while others highlight the importance of technical proficiency (13). Other analyses indicate that aerobic efficiency in muscular energy production is decisive for success over middle and long distances (14). Research has shown that VO₂max levels in young swimmers, alongside biomechanical (functional) variables, are most strongly associated with performance in the 400 m front crawl (4). Cardiopulmonary efficiency, oxygen consumption, and energy production are greater in young athletes at more advanced stages of biological maturation compared to their less developed peers, with differences ranging from 0.2 to 1.0 L·min⁻^1^. It has also been demonstrated that anaerobic capacity is less developed in young swimmers than in adults, highlighting the important role of biological maturation in anaerobic performance (15). Other authors, studying children aged 11–13 years, have argued that stroke length is the most important determinant of 400 m front crawl performance (8).

These findings illustrate the diversity of views regarding the impact of different variables on 400 m front crawl performance in prepubescent swimmers. Moreover, in most studies the experimental groups were formed through preliminary selection for swimming, which complicates the interpretation of results. This pre-selection tends to favour children who are tall, with long limbs, a long torso, large feet, and good aerobic capacity—traits already known to be advantageous in swimming. In the present study the participants began swimming voluntarily, without any prior selection on anatomical or physiological grounds. The results may therefore provide a more accurate picture of the impact of training and natural biological development on performance, independent of pre-selection. This addresses an important research gap that highlights the need for longitudinal studies of these processes. The aim of the present study is to identify which of the selected variables most strongly influence 400 m front crawl performance in prepubescent girls, using a longitudinal design. On the basis of the identified research gap, the study addressed the following questions: (1) Which variables are the strongest predictors of 400 m front crawl performance in prepubescent, non-elite female swimmers? And (2)How do these variables change over the course of a three-year training period? From these questions, two hypotheses were formulated: (1) Aerobic capacity will show the strongest influence on 400 m front crawl performance and (2) The examined variables will undergo significant changes over the three year period of observation. Answering these questions will make it possible to assess whether early pre-selection for swimming training in prepubescent girls is justified and to describe the developmental trajectories of anthropometric, physiological, and respiratory characteristics in girls aged 10–12 years.

## 2 Materials and methods

### 2.1 Sample

The study included 14 adolescent female swimmers (mean biological age at baseline: 10.52 ± 0.37 years; mean body mass: 34.99 ± 2.77 kg; mean height: 146.00 ± 3.05 cm), all training at student sports clubs in Częstochowa, Poland. Eligibility criteria were as follows: age of 10 years at study entry, commencement of structured swimming training at a student sports club in the same year, medical certification confirming the absence of contraindications to swimming (approved by the bioethics committee), and provision of written informed consent by legal guardians. Recruitment to the clubs was carried out without prior selection. At the beginning of the study, participants commenced formal swimming training, having previously acquired basic swimming skills during biweekly lessons. According to reports from their legal guardians, none of the participants engaged in additional sports activities beyond compulsory school physical education. Over the three years of the study, random events such as extended absences from training (over 2 weeks), health conditions, changes in residence, or inability to perform measurements excluded some participants from the experiment. The final number of participants is made up of people who participated in all measurement sessions (complete results) and completed at least 33 weeks of training in each cycle. All participants remained in the premenstrual stage throughout the six phases of data collection. Based on the Participant Classification Framework proposed by McKay et al. (16), the cohort can be categorised as Tier 2: Trained/Developmental. The study adhered to the principles of the Declaration of Helsinki. Participants and their parents were fully informed of the study's aims and procedures, and written consent was obtained from both participants and legal guardians. Ethical approval for the protocol was granted by the Bioethics Committee for Scientific Research at Jan Dlugosz University in Częstochowa (approval number KB-2/2012).

### 2.2 Longitudinal study design

**Figure 1:**
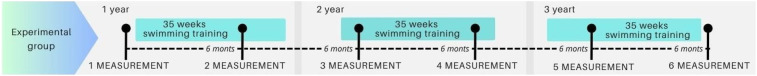
Study design diagram.

This was an experimental longitudinal study conducted over three consecutive years, from autumn 2011 to spring 2014. Measurements were performed at six-month intervals, between 8 AM and 12 PM, resulting in a total of six assessment points (Figure 1).

**Figure 2:**
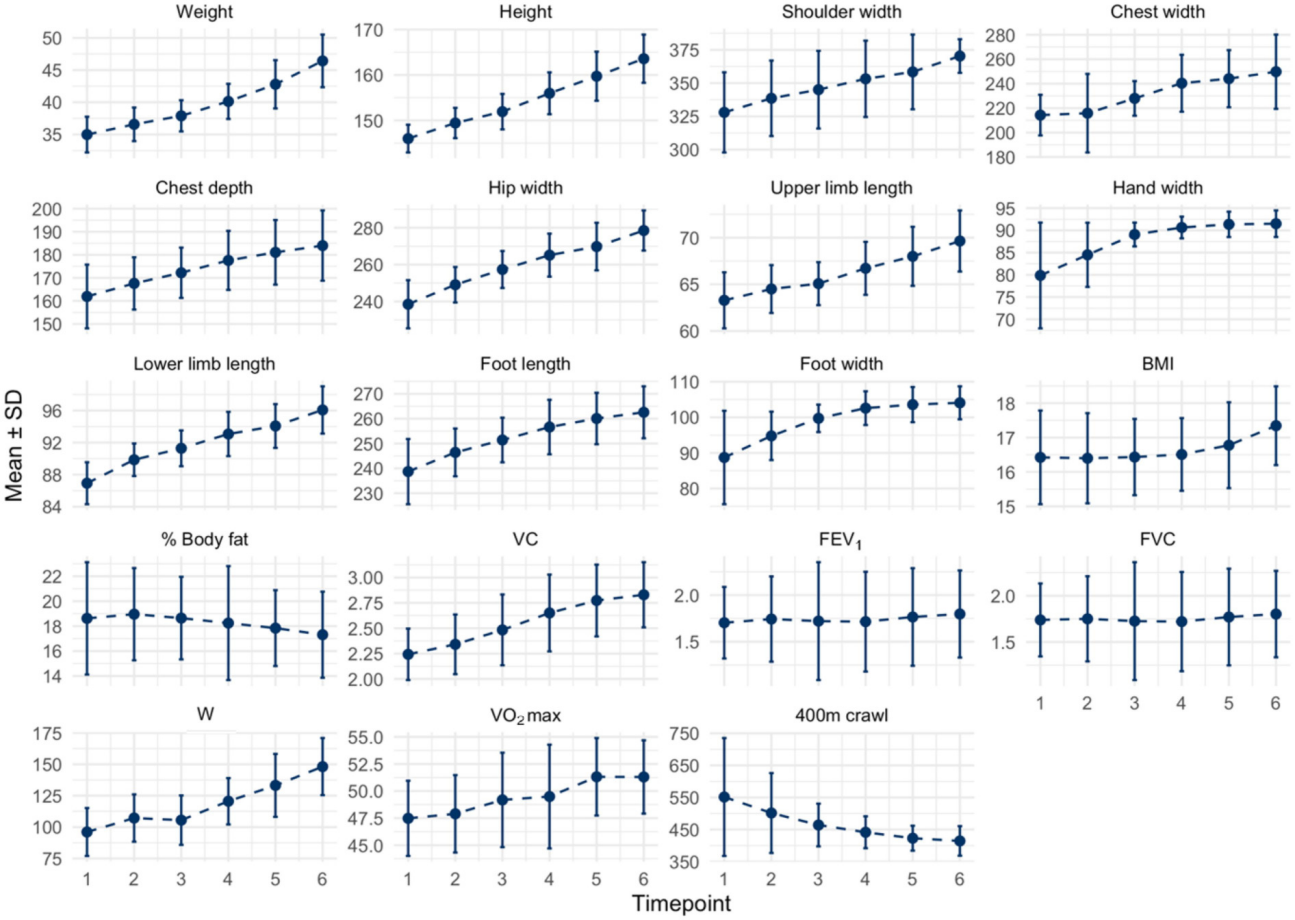
Training macrocycle of female swimmers (aged 9–12), according to British swimming federation guidelines.

The swimmers’ training macrocycle was designed in accordance with the British Swimming Federation guidelines for girls aged 9–12 years (17). Training sessions were held four times per week in the morning (06:30 AM–07:40 AM), each lasting 70 min and comprising a series of structured swimming drills (Figure 2). The average daily training distance increased progressively over the course of the study: approximately 1,500 metres in the first year, 2,000 metres in the second year, and 2,500 metres in the third year. Each session commenced with a 10-minute land-based warm-up, followed by a water-based warm-up covering 200–400 metres using front crawl or backstroke. Training focused on maintaining correct body position, optimising arm stroke and leg movement efficiency, and preserving correct swimming technique throughout the exercises.

Emphasis during training was placed on body position, the efficiency of arm strokes and leg movements, and the consistent maintenance of correct swimming technique. Attention was also given to improving turns and underwater phases. To support technical development, coaches incorporated the use of specialised equipment, including short and long fins, swim paddles, and resistance bands. Aerobic capacity was primarily developed through front crawl swimming. Each session concluded with approximately seven minutes of land-based stretching exercises, designed to enhance shoulder girdle mobility and increase ankle joint flexibility.

### 2.3 Biological age and antropometric mesurements

Body mass and height were measured using a scale with a stadiometer (WPT 150.0; RadWag, Poland) with an accuracy of 0.1 kg and 0.5 cm, respectively. Biological age was calculated using the formula described by Przewęda (18, 7), where body mass age and height age were estimated from Pirquet's tables for females from the Lubusz region (19), and according to Jopkiewicz 1998 (20).

Biologicalage=(bodymassage+bodyheightage+chronologicalage)/3

Body mass/hight age−estimated using Pirquet’s tables

Chronological age−time between date of birth and the date of measurement

The following anthropometric measurements were taken: body mass; height (B–v); chest depth (xi–ths); chest width (thl–thl); shoulder width (a–a); hip width (ic–ic); upper limb length (a–da); hand width (mu–mr); lower limb length (B–sy); foot width (mtt–mtf); and foot length (pte–ap) (21). Measurements were performed on the right side of the body in a standing position (Frankfurt plane) using a spreading caliper with 1 mm precision. Body mass index (BMI) was calculated as body mass (kg) divided by height squared (m^2^). All assessments were conducted between 8:00 AM and 12:00 PM.

### 2.4 Body fat measurements

Body fat percentage was assessed by measuring skinfold thickness at four anatomical sites: over the biceps, over the triceps, beneath the inferior angle of the scapula (shoulder blade), and above the iliac crest (abdomen). All measurements were taken on the right side of the body in a standing position (Frankfurt plane) using a Harpenden skinfold caliper (M2 TOP, Käfer, Germany) with 0.1 mm precision. Body fat percentage was calculated according to the formula by Slaughter et al. (22). For participants whose sum of triceps and subscapular skinfolds was ≤35 mm, the following equation was used:

$$\text{\%}\mathrm{Body}\;\mathrm{fat}=[1.33\times (R+L)]-[0.013\times (R+L{)}^{2}]-2.5$$

R = skinfold over the triceps

L = skinfold beneath the scapula

For participants with a sum >35 mm, the equation was:

%Bodyfat=0.546×(R+L)+9.7

R = skinfold over the triceps

L = skinfold beneath the scapula

### 2.5 Aerobic and anaerobic capacity measurements

Aerobic capacity was measured using the Maximal Multistage 20 m Shuttle Run Test (Beep Test) (23). Participants ran back and forth over a 20 m distance, paced by audio signals that progressively shortened the allowed time. The initial running speed was 8.5 km/h, increasing by 0.5 km/h at each stage. The number of shuttle runs also increased with each stage: seven runs in stage one, eight in stage two, ten runs from stages five to eight, and twelve runs from stages nine to thirteen. Participants were required to reach the line before the next signal; failure to do so ended the test. The total number of successful shuttle runs was recorded. Maximal oxygen uptake (VO₂max), an indicator of aerobic fitness, was calculated using the participant's maximum running speed at the last completed stage (P) and chronological age (W) with the formula by Léger et al. (24):

$$\dot{V}O_{2}\max=31.025+3.238\times P-3.248\times W+0.1536\times P\times W$$

P = maximum running speed (km/h) from the last completed stage

W = chronological age, rounded down to the nearest whole number

Anaerobic capacity was evaluated using the Standing Reach Jump test. The participant stood sideways (based on dominant hand) against a wall, arm fully extended upward, and the reach height was marked. They then performed a vertical jump with knees bent at 90° and an arm swing, marking the highest point reached. The test was performed three times without shoes, and the best result was used. The mechanical work (W) was culated from the jump height (h), body mass (m), and gravitational acceleration (g = 9.81 m/s^2^) as follows (25).

W=m×g×h

m = body mass in kilograms

g = 9.81 m/s^2^

h = jump height in meters

This value reflects the ability of the lower limb muscle to generate energy over a very short time period. Because the execution of a vertical jump lasts less than one second, the primary energy source is the phosphagen system. Therefore, the mechanical work derived from the jump can be considered an indicator of maximal anaerobic work (26, 27).

### 2.6 Respiratory measurements

Respiratory volumes were measured using a VF-S spirometer (PELAB, Poland), including vital capacity (VC), forced vital capacity (FVC), and forced expiratory volume in the first second (FEV_1_). For VC, participants sat and breathed calmly for several minutes before standing and taking the deepest possible inhalation, followed by maximal exhalation into the spirometer for at least six seconds, with a nose clip applied. The test was repeated three times with 5-minute intervals, and the best result was recorded. FEV_1_ was measured using a similar procedure, but participants performed a rapid, forceful exhalation, expelling as much air as possible within one second after assuming a standing position. FVC, representing the total volume of air forcefully exhaled after maximal inhalation, was measured by having participants take a deep breath while standing and then exhale as quickly and completely as possible into the mouthpiece.

### 2.7 Statistical analysis

The study included a relatively small sample of 14 subjects. *post hoc* power analysis using G*Power software (version 3.1.9.2; University of Cologne, Germany) indicated that a minimum of 12 measurements was required for *α* = 0.05, effect size *f* = 0.6, and *β* = 0.95. Participants diets were not controlled or recorded. Aerobic capacity was assessed indirectly via a running test due to the absence of consent from legal guardians for direct measurement. Menstrual age was determined based on information provided by the participants' legal guardians. All statistical analyses were conducted in R (version 4.0.4) (28). Descriptive statistics, including means and standard deviations, were calculated for all anthropometric, physiological, and respiratory variables across six measurement points. Longitudinal changes were evaluated using repeated measures ANOVA to identify statistically significant differences between initial and final time points. Normality was assessed using the skewness statistic, with a cutoff range of −1 to +1. In addition to significance testing, effect sizes were calculated to evaluate the magnitude of group differences. Eta^2^ was derived from ANOVA models as a measure of explained variance, and Cohen's d was computed to quantify standardized mean differences between baseline and final assessments. To examine associations between swimming performance (400 m crawl) and selected variables, Pearson correlation coefficients were calculated for each individual predictor, using mean scores calculated across the 6 measurement timepoints.

### 2.8 Multivariable prediction of 400 m crawl

To examine predictors of 400 m crawl performance, we employed generalized estimating equations (GEE), an approach well-suited to repeated-measures data with correlated outcomes. In our study, 14 swimmers were assessed on six occasions, which naturally introduced non-independence among observations. GEE models address this by estimating population-averaged effects while allowing for within-individual correlation (29). Predictors were decomposed into within-swimmer centred values, representing session-specific deviations from an individual's mean, and between-swimmer means, defined as the overall average across sessions for each swimmer. This decomposition allowed us to distinguish intra-individual effects, capturing how fluctuations around an athlete's usual value related to performance, from inter-individual effects, reflecting differences between athletes' overall profiles. The general model took the form:

$$\mathrm{CrawlScor}e_{it}=\;\beta 0\;+\;\Sigma \;\beta_{\mathrm{within}}(X_{itj}-\;{\bar{X}}_{ij})+\;\Sigma \;\beta_{\mathrm{between}}{\bar{X}}_{ij}+\;\varepsilon_{it}$$

Where *i* denotes the athlete (cluster), *t* the measurement occasion, and *j* the predictor (e.g., weight, height, VO₂max). CrawlScore_it_ is the 400 m crawl time for swimmer *i* at time *t*. *β*₀ is the intercept, *β*_within_ represents the effect of within-swimmer deviations from their own mean, and *β*_between_ represents the effect of between-swimmer differences in average predictor values. *X_itj_* is the value of predictor *j* for swimmer *i* at time *t*, *X¯_ij_* is the mean of predictor *j* for swimmer *i* across all timepoints, and *ε_it_* is the residual error term.

The outcome variable, 400 m crawl time, was treated as continuous, assumed to follow an approximately normal distribution, and modelled with an identity link so that predictions corresponded directly to performance times. Because assessments were repeated over time, we specified a first-order autoregressive correlation structure [AR(1)], which assumes that observations closer in time are more strongly correlated than those further apart. This choice is appropriate for longitudinal designs such as ours, in which swim performances within the same season are expected to be more similar than those measured at longer intervals. Model adequacy and correlation structure selection were guided by the quasi-likelihood under the independence model criterion (QIC), which provides a measure of relative model fit for GEE models (30). QIC is widely recognised as the preferred approach for comparing alternative working correlation structures in repeated-measures data, including independence, exchangeable, and AR(1) specifications (31, 32). In our analyses, the AR(1) structure consistently produced the lowest QIC values, providing empirical justification for its adoption.

Because many predictors were intercorrelated, we assessed multicollinearity using both variance inflation factors (VIF) and pairwise correlations. To evaluate robustness, we estimated three model specifications: a full model including all predictors; a VIF-screened model excluding variables with VIF ≥ 5; and a correlation-screened model excluding one variable from each highly correlated pair (|*r*| ≥ 0.80), prioritising those with lower average correlations. Model diagnostics, including residual plots and influence analyses, were performed to assess adequacy, and sensitivity to individual participants was examined using a leave-one-cluster-out (LOCO) procedure, in which the GEE was re-estimated after excluding each swimmer in turn and the maximum absolute change in any regression coefficient was recorded.

## 3 Results

### 3.1 Descriptive statistics

**Figure 3:**
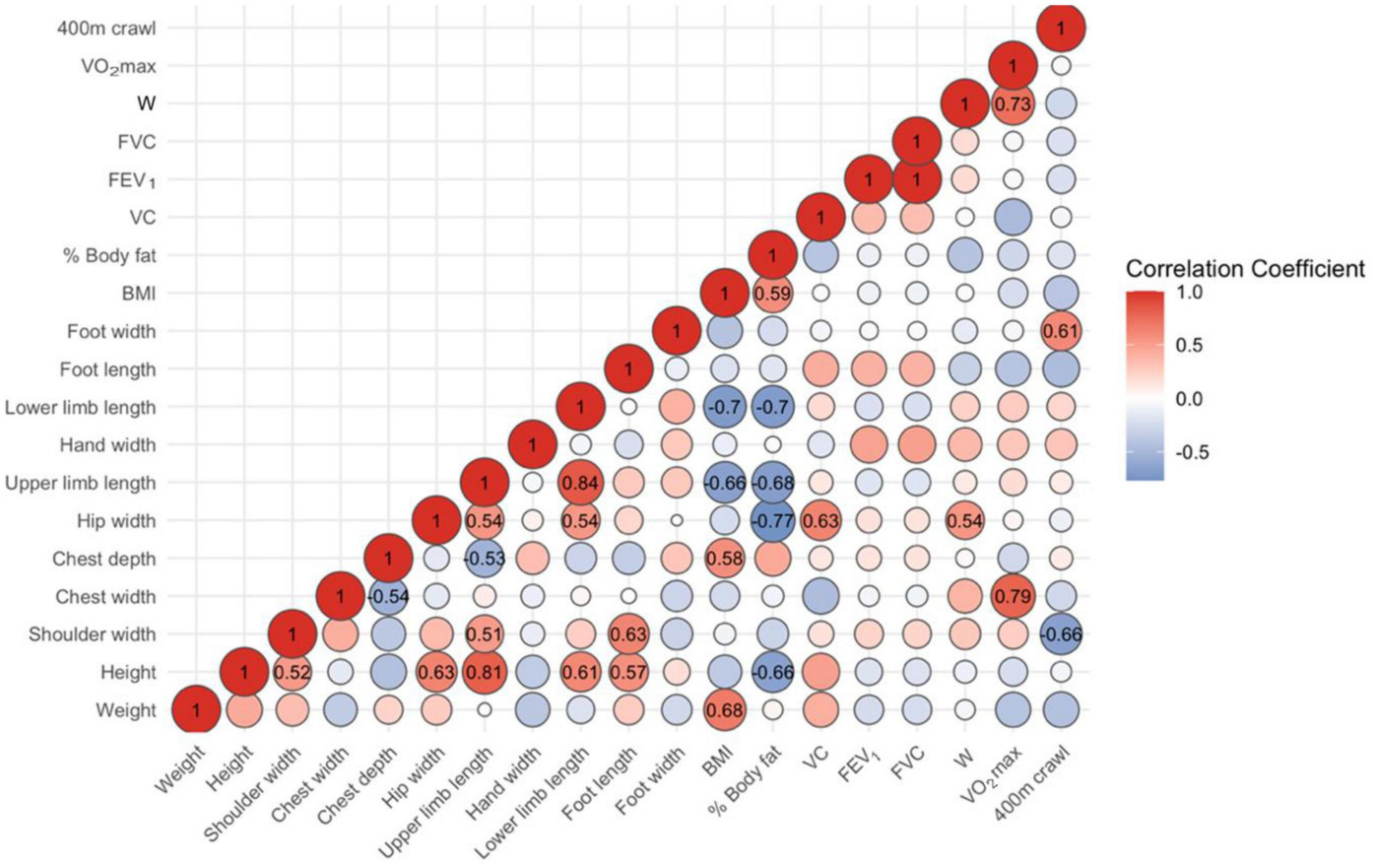
Trends in anthropometric, physiological, and respiratory metrics across six follow-up assessments. SD, standard deviation; BMI, body mass index; VC, vital capacity; FEV_1_, forced expiratory volume in one second; FVC, forced vital capacity; W, work of lower limbs; VO_2_max, maximal oxygen uptake. All anthropometric measurements are reported in millimeters, except for upper and lower limb lengths, which are given in centimeters.

Table 1 provides descriptive and inferential statistics for anthropometric, physiological, and performance variables across six measurement time points. Skewness and kurtosis values indicated that distributions were approximately normal, with no evidence of severe skew. Variable distributions are illustrated in Supplementary Figure S1. Figure 3 illustrates mean trends across the study period. Individual swimmer trajectories are presented in Supplementary Figure S2. ANOVA tests comparing baseline and final measurement revealed statistically significant improvements in most anthropometric and performance variables. No significant differences were observed for BMI, % body fat, FEV_1_, or FVC. Most anthropometric differences were large. Height (*d* = 4.06), weight (*d* = 3.28), hip width (*d* = 3.33), and lower limb length (*d* = 3.28) showed the largest effects. Moderate-to-large effects were also found for chest width (*d* = 1.45), chest depth (*d* = 1.52), and foot dimensions (*d* = 2.01–1.56). Physiological measures revealed similarly large effects for VC (*d* = 2.03), W (*d* = 2.48), and VO₂max (*d* = 1.11).

**Table 1:** Descriptive statistics and ANOVA results comparing the initial and final measurements.

Variable	Mean	SD	Median	Min	Max	Range	Skew	Kurtosis	F-value	Df	*P*-value	Eta2	Cohen's d
Weight (kg)	39.93	2.74	38.68	35.80	44.23	8.43	0.33	−1.35	75.18	1.00	0.00	0.74	3.28
Height (cm)	154.79	4.11	154.67	149.25	164.83	15.58	0.81	0.32	115.52	1.00	0.00	0.82	4.06
Shoulder width (mm)	351.36	18.07	357.67	317.50	373.50	56.00	−0.52	−1.21	23.53	1.00	0.00	0.48	1.83
Chest width (mm)	231.58	19.55	224.17	208.33	279.83	71.50	1.05	0.26	14.72	1.00	0.00	0.36	1.45
Chest depth (mm)	174.17	12.29	175.83	149.83	195.67	45.83	−0.32	−0.48	16.12	1.00	0.00	0.38	1.52
Hip width (mm)	260.45	8.16	260.17	250.33	274.17	23.83	0.26	−1.47	77.47	1.00	0.00	0.75	3.33
Upper limb length (cm)	66.46	2.60	66.50	62.50	71.17	8.67	0.00	−1.09	28.72	1.00	0.00	0.52	2.03
Hand width (mm)	88.02	2.90	86.50	84.50	92.50	8.00	0.39	−1.60	12.62	1.00	0.00	0.33	1.34
Lower limb length (cm)	92.03	2.27	92.17	88.67	96.67	8.00	0.20	−0.75	75.37	1.00	0.00	0.74	3.28
Foot length (mm)	253.44	8.27	250.50	242.25	265.67	23.42	0.17	−1.80	28.32	1.00	0.00	0.52	2.01
Foot width (mm)	98.79	3.12	98.33	94.33	104.83	10.50	0.43	−1.01	17.06	1.00	0.00	0.40	1.56
BMI (kg/m^2^)	16.62	1.10	16.42	14.82	18.65	3.83	0.19	−0.92	3.75	1.00	0.06	0.13	0.73
Body fat (%)	18.18	2.97	17.94	13.47	24.17	10.70	0.23	−0.75	0.74	1.00	0.40	0.03	−0.33
VC (l)	2.57	0.31	2.56	2.07	3.12	1.04	0.11	−1.21	28.81	1.00	0.00	0.53	2.03
FEV_1_ (l)	1.76	0.47	1.73	0.99	2.50	1.51	0.10	−1.36	0.34	1.00	0.57	0.01	0.22
FVC (l)	1.77	0.47	1.73	1.00	2.51	1.50	0.08	−1.37	0.15	1.00	0.70	0.01	0.15
W (J)	118.88	17.96	119.88	90.25	150.64	60.39	−0.06	−1.14	43.16	1.00	0.00	0.62	2.48
VO_2_max (ml*min^−1^*kg^−1^)	49.39	3.31	49.72	42.13	54.47	12.35	−0.63	−0.29	8.69	1.00	0.01	0.25	1.11
400 m crawl (s)	461.57	80.65	430.49	362.87	612.22	249.35	0.68	−0.98	7.32	1.00	0.01	0.22	−1.02

SD, standard deviation; df, degrees of freedom; BMI, body mass index; VC, vital capacity; FEV_1_, forced expiratory volume in one second; FVC, forced vital capacity; W, work of lower limbs; VO_2_max, maximal oxygen uptake.

### 3.2 Multivariable prediction of 400 m crawl score

**Figure 4:**
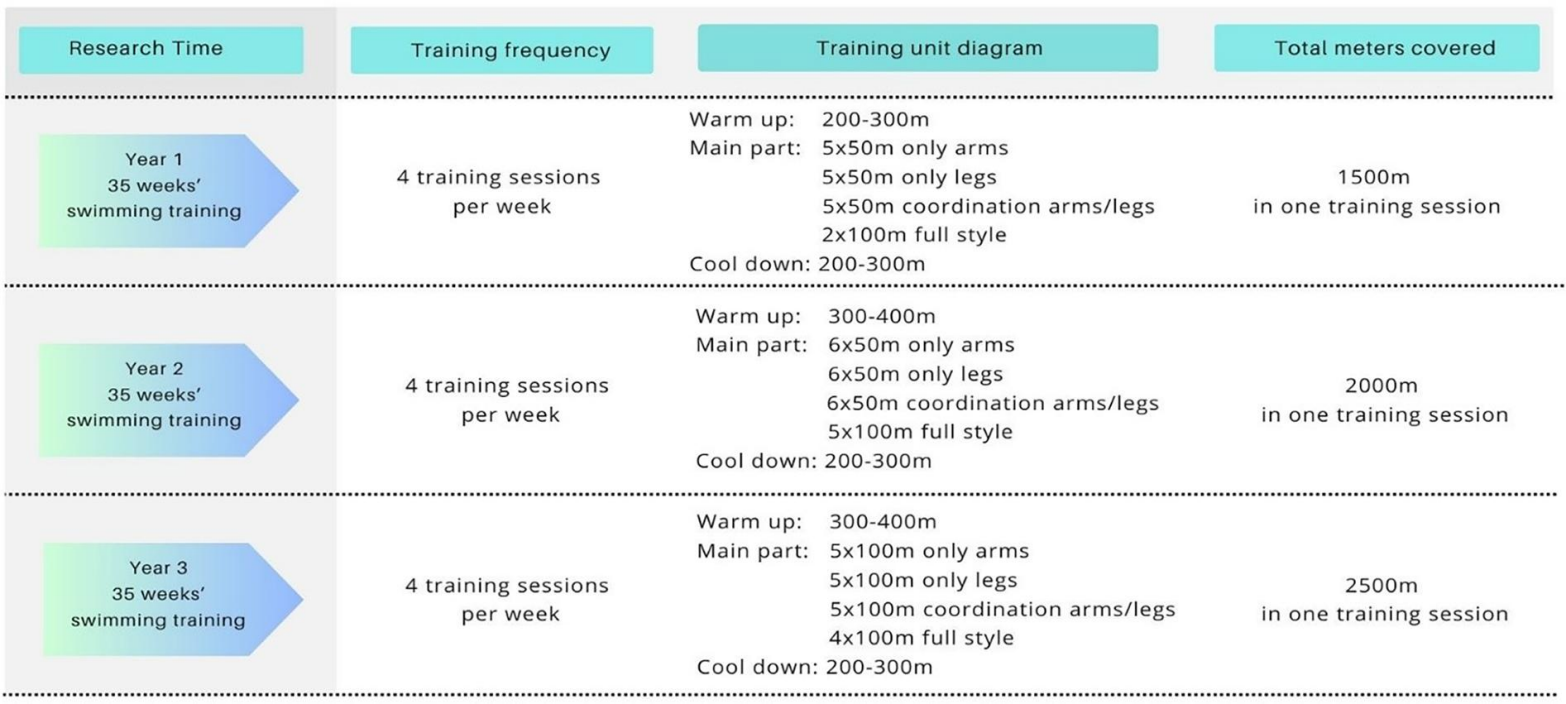
Heatmap of correlation coefficients between anthropometric, physiological, and respiratory measures and 400 m crawl score. BMI, body mass index; VC, vital capacity; FEV_1_, forced expiratory volume in one second; FVC, forced vital capacity; W, work of lower limbs; VO_2_max, maximal oxygen uptake.

Correlation analyses indicated that 400 m crawl performance (lower times = better performance) was most strongly associated with anthropometric variables (Figure 4). In particular, shoulder width (*r* = –0.66) and foot length (*r* = –0.46) showed moderate-to-strong negative correlations, suggesting broader shoulders and longer feet were linked to faster swim times. Body mass also correlated negatively with crawl time (*r* = –0.43), while BMI demonstrated a moderate association (*r* = –0.39). In contrast, foot width correlated positively (*r* = 0.61), indicating greater width was linked to slower performance. Other anthropometric measures, including height and hip width, showed only weak or negligible relationships. Body fat percentage was only weakly related (*r* = –0.19). Physiological measures such as VO₂max (*r* = –0.04) and VC (*r* = –0.07) were essentially unrelated, and respiratory indices (FEV_1_ and FVC, both *r* ≈ −0.21) showed weak negative correlations. External mechanical work performed during a vertical jump demonstrated a modest negative association (*r* = –0.27).

Generalized estimating equation models were used to evaluate anthropometric and physiological predictors of 400 m crawl time. Model fit varied across specifications. Across all models, residuals aligned closely with the 45° reference line, indicating approximate normality. Minor deviations were observed in the tails, but no major violations of distributional assumptions were detected (Supplementary Figure S3). Residuals were generally symmetrically distributed around zero with no strong evidence of systematic patterns, suggesting that model assumptions of linearity and homoscedasticity were reasonably met. Minor heteroscedasticity was observed at extreme fitted values but did not substantially affect model adequacy (Supplementary Figure S4). The influential cases analysis revealed that a small number of participants (3 swimmers) produced large changes in regression coefficients when excluded, indicating disproportionate influence on the model, while most subjects had minimal impact (Supplementary Figure S3). Model diagnostics, including residual plots, are presented in Supplementary Figures S4, S5. The full model including all 18 predictors explained 55% of the variance. After screening for multicollinearity, explanatory power decreased: the VIF-screened model with 7 predictors explained 28% of the variance, while the correlation-screened model with 14 predictors explained 46%. In the full model, greater BMI (*β* = −211.0; 95% CIs = −384.9, −37.1; *p* = 0.02) and weight (*β* = 71.0; 95% CIs = 7.2, 134.8; *p* = 0.03) were both associated with crawl times, whereas longer foot length (*β* = −6.18; 95% CIs = −9.73, −2.63; *p* = 0.001) was associated with faster performance. Both BMI and weight were significant predictors but with coefficients in opposite directions. This reflects the strong collinearity between these measures, since BMI is derived from measures of weight and height. The coefficients therefore represent conditional effects (e.g., BMI adjusted for weight, and weight adjusted for BMI) rather than independent contributions. In the VIF-screened model, two physiological predictors remained significant, including higher VC (*β* = −73.0; 95% CIs = −136.3, −9.7; *p* = 0.02) and lower % body fat (*β* = −8.22; 95% CIs = −16.0, −0.44; *p* = 0.04) (Supplementary Figure S5). In the correlation-screened model, two foot measures were retained as significant predictors. Longer foot length (*β* = −6.01; 95% CIs = −8.97, −2.15; *p* = 0.002) predicted better crawl performance, while greater foot width (*β* = 5.62; 95% CIs = 1.52, 9.72; *p* = 0.01) predicted slower performance (Supplementary Figure S5). While the exact set of predictors varied depending on how multicollinearity was addressed, foot dimensions consistently contributed to crawl performance. Although several predictors reached conventional significance thresholds, the wide confidence intervals highlight substantial uncertainty around the precise effect sizes; our interpretation therefore emphasizes the direction and magnitude of associations, rather than focusing narrowly on *p*-values.

## 4 Discussion

The research hypothesis of this project assumed that aerobic capacity would be the most important determinant of 400 m front crawl performance. However, the results of this longitudinal study indicate that foot length, vital capacity (VC), and body fat percentage play the most important roles in determining 400 m freestyle performance in prepubescent girls participating in swimming training. Foot length, particularly when accompanied by relatively small width, was shown to have a positive effect on 400 m front crawl performance. This is consistent with reports from other researchers who emphasise that appropriate foot length, combined with reduced width, enhances the generation of propulsive force by the lower limbs, especially during the underwater phase following the push-off from the turn wall (33). During distance swimming, particularly in the front crawl, effective foot action supported by greater foot length generates stronger propulsion for the swimmer (8). Furthermore, greater foot length plays an important role in body alignment in the water. Our results suggest that longer feet, when used efficiently, contribute to improved positioning of the head, shoulders, and hips at the water surface, resulting in higher swimming velocity (34). A long foot in swimmers also contributes to increased propulsive force, especially in alternating strokes. In swimming, forward motion results from the action of different forces. Dynamic swimming may be generated both by external forces, such as wall push-offs, and by limb movements that initiate forward propulsion. Just as static swimming involves the interaction of gravity and buoyancy, dynamic swimming involves hydrodynamic reactions with a horizontal component that alters the balance of forces (35, 36). Propulsive force in swimming is the outcome of the interaction between a swimmer's limbs and the aquatic environment, expressed as water resistance reacting to limb movements. This force results in forward displacement of the swimmer. To accelerate the body, propulsive force must have a point of application, a direction, magnitude, and be generated with sufficient strength from both upper and lower limbs (35). As research and observation indicate, the rhythm of leg movements is primarily determined by the swimming stroke, with distinct patterns observed in breaststroke compared with front crawl (36). A swimmer with excellent movement coordination is characterised by minimal acceleration and deceleration phases within a single cycle (37). This is largely dependent on leg action. Observations of swimmers reveal that they do not move at a constant velocity but rather experience variations in acceleration across different phases of the cycle. These changes are influenced by body mass, water density and temperature, and the time interval between maximum and minimum velocity, which in turn depends on the movement cycle. Propulsive force is most effective when the swimmer maintains forward motion with minimal variations in acceleration. In the front crawl, continuous alternating leg movements allow for easier maintenance of steady acceleration; hence, longer feet exert a significant effect on sustaining consistent velocity over the race distance (37). The second most important factor predicting results in the 400 m freestyle was vital capacity (VC). Researchers agree that swimmers display superior respiratory parameters compared with their non-swimming peers (38). This is associated with long-duration exercise in the horizontal position of the chest, exposed to variable hydrostatic pressure. It also involves periods of apnoea, leading to brief but regularly repeated hypoxic states (39) Most studies investigating the influence of swimming training on respiratory parameters in prepubescent children demonstrate increased forced vital capacity in swimmers compared with non-swimmers and children engaged in other sports, which is consistent with the results of our study (40). These differences are evident even when body height is comparable. It has also been shown that swimmers exhibit increased dead space, stimulating the body to greater breathing depth and frequency (41). Pulmonary vital capacity is often higher at the outset of training due to the tendency to select taller individuals for swimming. Furthermore, it has been demonstrated that one year of intensive endurance swimming training leads to significant increases in both static and dynamic lung volumes, with particular improvement in flow–volume relationships among prepubescent female swimmers (42). These findings suggest that respiratory muscles can gain strength and endurance in response to specific aquatic training conditions, such as restricted expiration into water. Such adaptations may be explained by five water-related stressors: (i) breathing under pressure and prolonged expiration into water, (ii) restricted breathing patterns and altered alveolar gas tensions, (iii) repeated expansion of the lungs to total lung capacity, (iv) prone body position, and (v) intensive training programmes initiated in early childhood. As a result, minute ventilation is reduced and swimmers' lungs must accommodate large gradients of CO₂ and O₂ partial pressures, leading to enhanced diffusing capacity (43). The high adaptability of healthy lungs, combined with the stronger response of younger individuals to growth stimuli, provides a strong explanation for the improved respiratory capacity of swimmers. Prolonged breath-holding, particularly during underwater phases after the start and turns, contributes to the development of respiratory muscles, including the diaphragm. Increased hydrostatic pressure during immersion improves chest wall elasticity, resulting in higher lung function (44). Importantly, in short-term studies lasting no longer than six months, authors did not observe significant changes in respiratory parameters in swimmers compared with non-swimmers and athletes from other sports (e.g., athletics, basketball, rowing), attributing this to the limited time for measurable adaptations to emerge (45). In other studies, comparing swimmers and football players with age- and sex-matched controls, swimmers were found to have significantly higher values of vital capacity and forced expiratory volume in one second. Moreover, the same researchers analysed the influence of training-related factors such as training duration, age of initiation, and weekly training frequency on pulmonary function in young swimmers (aged 10–13 years), controlling for anthropometric features. They concluded that none of these factors significantly influenced predicted spirometry values (46). Our results indicate that swimmers with superior respiratory parameters are better predisposed to perform better over long distances, due to their ability to remain underwater for longer periods after the start and during turns. The third determinant influencing the result in the 400-meter front crawl was the percentage of body fat. Based on our findings, it's safe to conclude that swimming may be an effective way to prevent obesity in early life, given its low injury rates and numerous health benefits. It's worth noting, however, that we didn't control the children's diet or other forms of physical activity during the experiment, which may influence the results, although a trend toward reduced body fat is evident in the experimental group. Reducing body fat percentage may also translate into improved athletic performance over specific distances and in selected swimming styles (47), as evidenced by the results of this study. Given the modest sample size (*N* = 14) relative to the number of predictors, the model prioritized parsimony, retaining only variables that contributed unique and robust predictive value. Many anthropometric variables, such as limb lengths and body mass indices, are highly intercorrelated; as a result, the model likely selected the most informative representative (e.g., foot length) while shrinking the coefficients of redundant variables to zero. Similarly, other physiological measures, such as VO₂max and Anaerobic Work, may have shared variance with included predictors or failed to provide sufficient additional predictive power within the constraints of the penalized regression. Thus, the exclusion of most variables should not be interpreted as evidence of their irrelevance, but rather as a reflection of the model's efficiency in balancing complexity and predictive accuracy under conditions of limited data.

## 5 Limitations

Several limitations of this study should be acknowledged. First, the sample size was relatively small (*N* = 14). Although *post hoc* analysis using G*Power software (version 3.1.9.2; University of Cologne, Germany) indicated that a minimum of 12 measurements would be sufficient for *α* = 0.05, effect size *f* = 0.6, and *β* = 0.95, the limited number of participants may nonetheless restrict the generalisability of the findings. Second, participants’ dietary habits were not monitored or controlled, which may have influenced body composition and physiological outcomes. Third, the children's aerobic capacity was measured indirectly using the 20-meter Shuttle Run Test. The authors did not obtain consent from their legal guardians for direct (laboratory) measurement. According to the scientific findings in the Mayorga-Vega meta-analysis (48) the beep test is a highly accurate alternative to measuring VO_2_max when laboratory measurement is not possible. This methodological constraint may have limited the accuracy of the aerobic capacity data in relation to swimming performance. Finally, menstrual age was determined via interviews with legal guardians rather than through direct clinical assessment, which may have introduced reporting bias. Finally, the predictive model demonstrated suboptimal fit, reflecting the challenges of balancing model complexity with statistical power in a dataset of this size.

## 6 Conclusions

The results of this experiment suggest that coaches and swimming instructors should approach the concept of pre-selection with caution. Although foot length was found to be one of the predictors of 400 m front crawl performance, other anthropometric parameters were not of primary importance. Furthermore, in our study aerobic capacity was not the most significant factor in improving performance, contrary to our initial assumptions. Nevertheless, it can be assumed that girls with superior respiratory parameters and greater foot length will achieve better results in long-distance front crawl compared with peers who do not possess these somatic and physiological traits. Further research with larger sample sizes and including dietary assessment is required to provide more comprehensive insights into other characteristics that may influence 400 m freestyle performance in girls. Conducting similar studies in older, post-pubescent groups will provide valuable information on how performance determinants change over time. Future research should also include the monitoring of front crawl technique using laboratory-based equipment, with particular emphasis on tethered swimming tests to measure propulsive force.

## Data Availability

The raw data supporting the conclusions of this article will be made available by the authors, without undue reservation.
