# Supplementary material for: Determinants of pre-adolescent girls’ sport performance in three-year swimming training
Source: Front Sports Act Living. 2025 Dec 3;7:1710646. doi: 10.3389/fspor.2025.1710646 (PMC12708920; doi:10.3389/fspor.2025.1710646)
Supplement: Supplementary file 1 [file Datasheet1.docx]

Supplementary Material

| 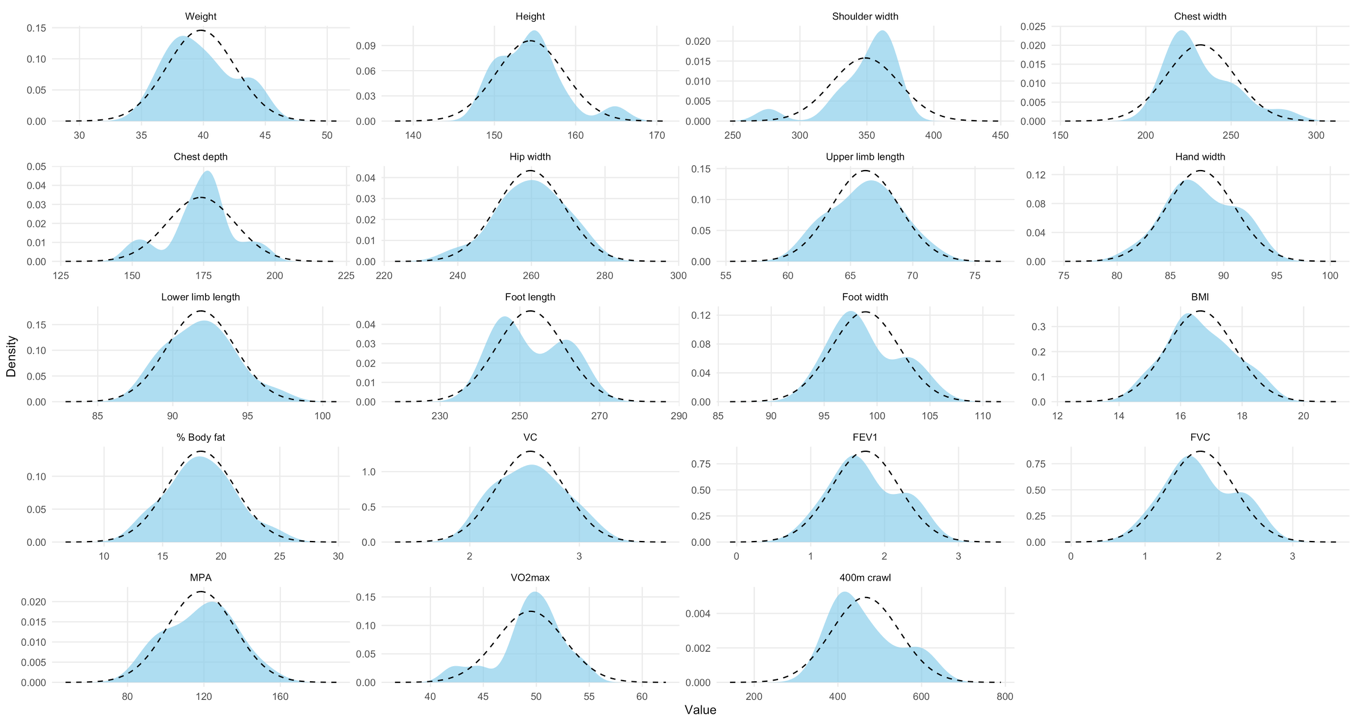 |
| --- |
| Supplementary Figure 1. Variable distributions. |

| 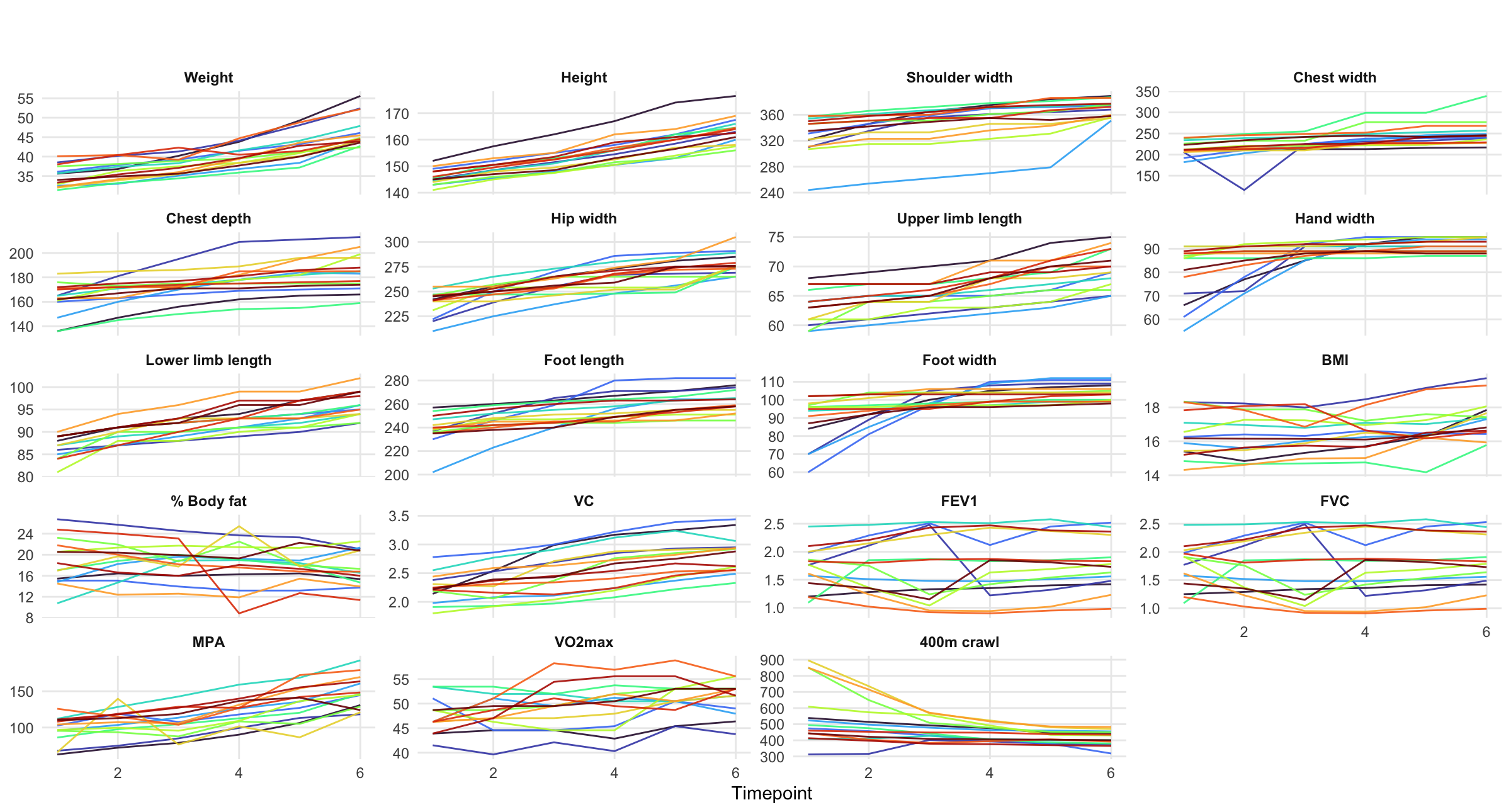 |
| --- |
| Suplementarny Figure 2. Individual swimmer trajectories throughout the study period. |

| 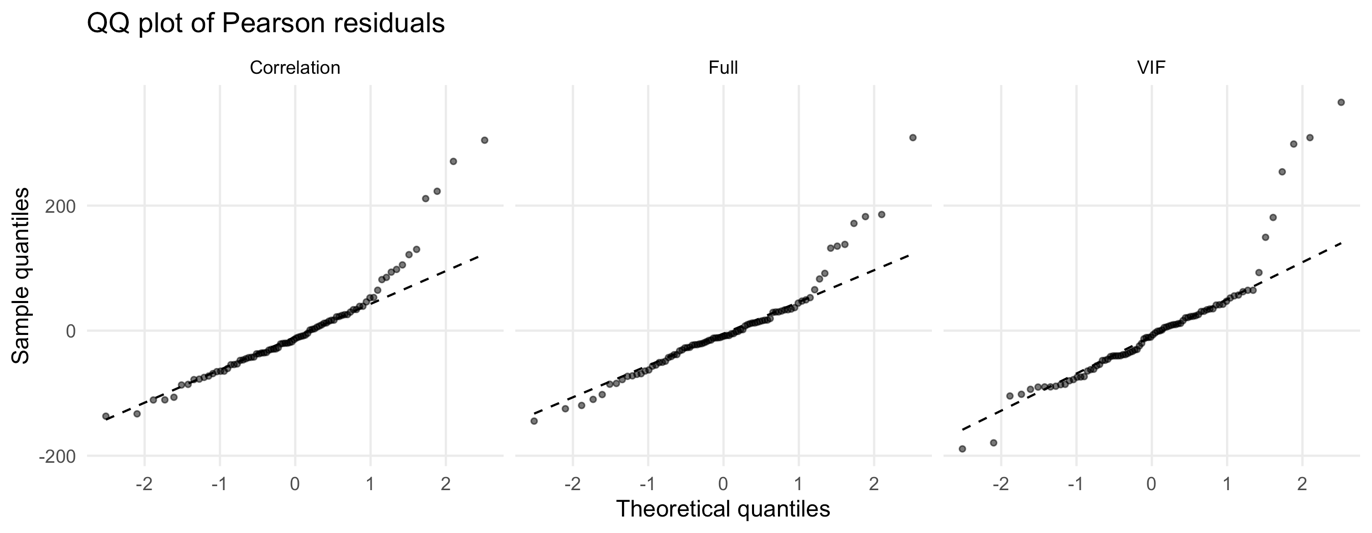 |
| --- |
| Supplementary Figure 3. Q–Q plots of model residuals for the three specifications (full model, VIF-screened model, and correlation-screened model).   \| 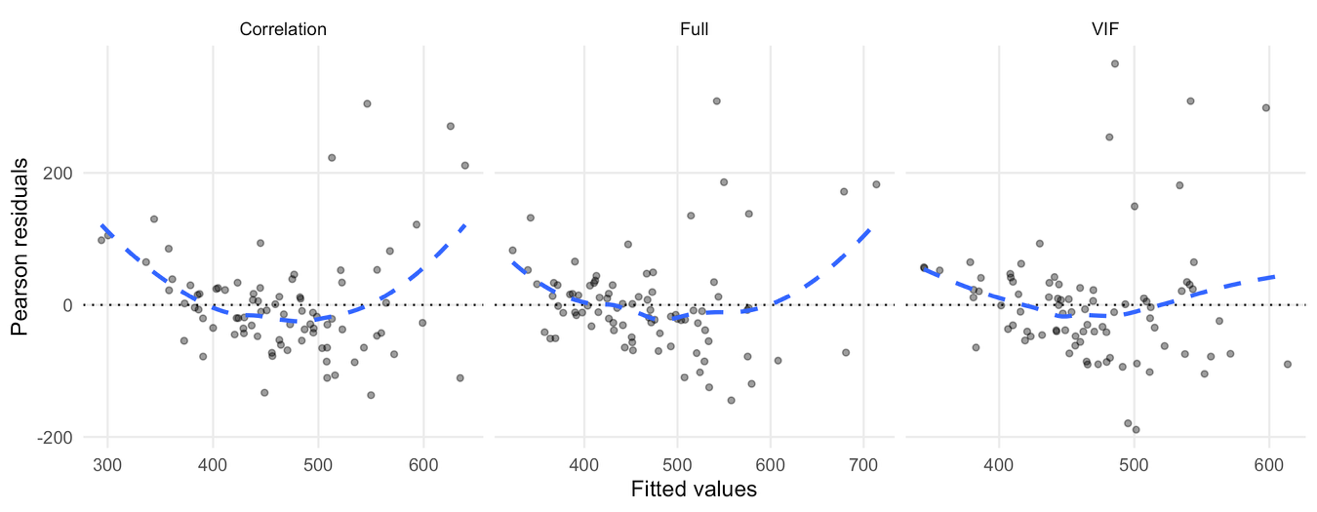 \| \| --- \| \| Supplementary Figure 4. Residuals versus fitted values for the three model specifications (full, VIF-screened, and correlation-screened). \| |

| 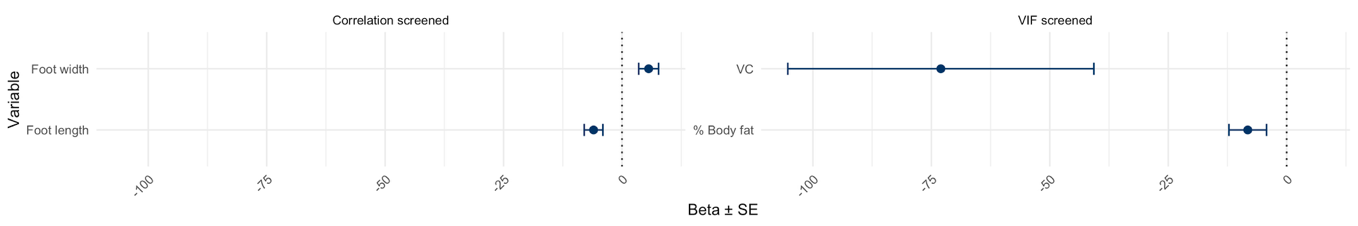 |
| --- |
| Supplementary Figure 5. Beta coefficients and standard errors. |
